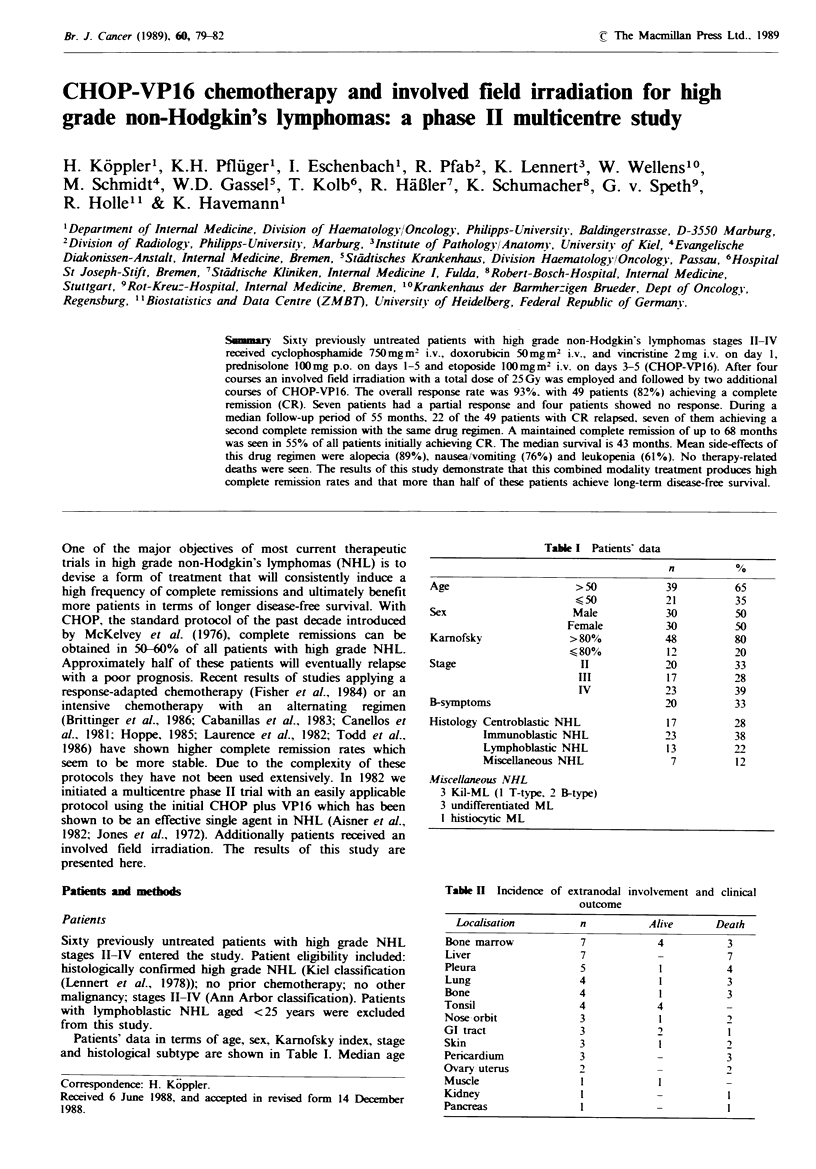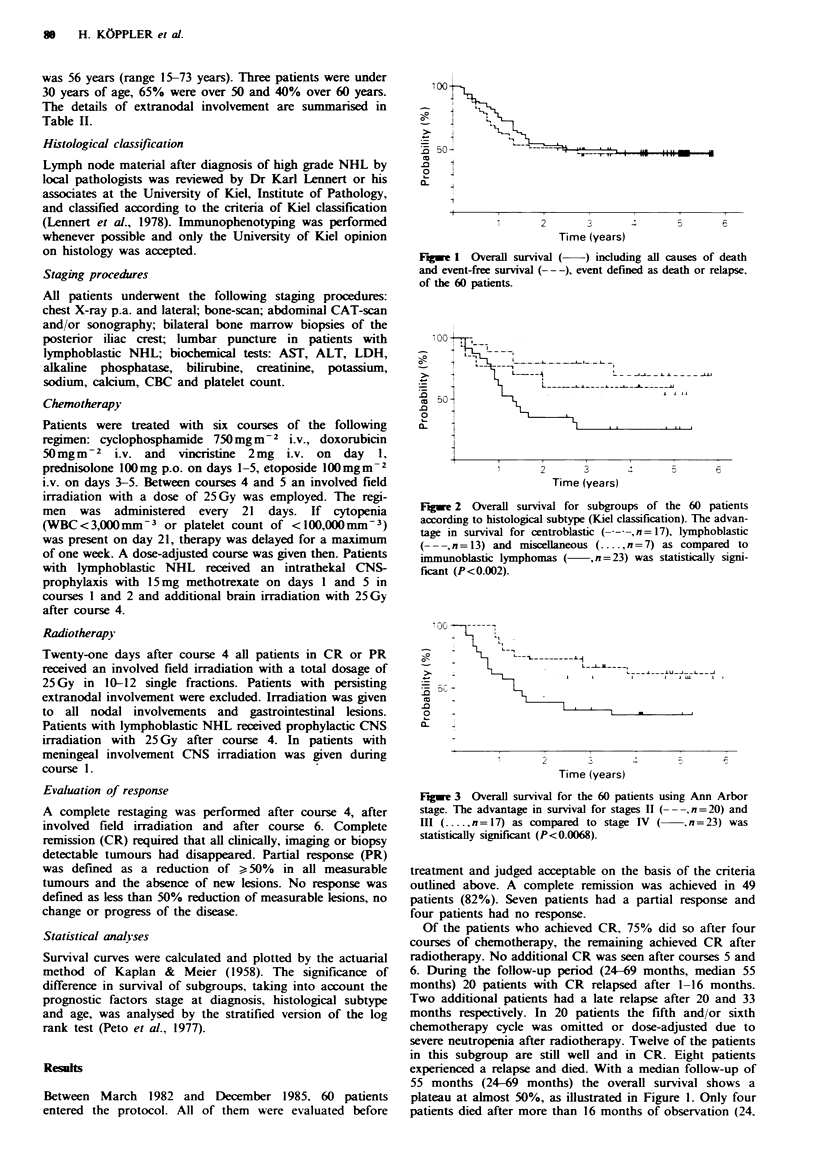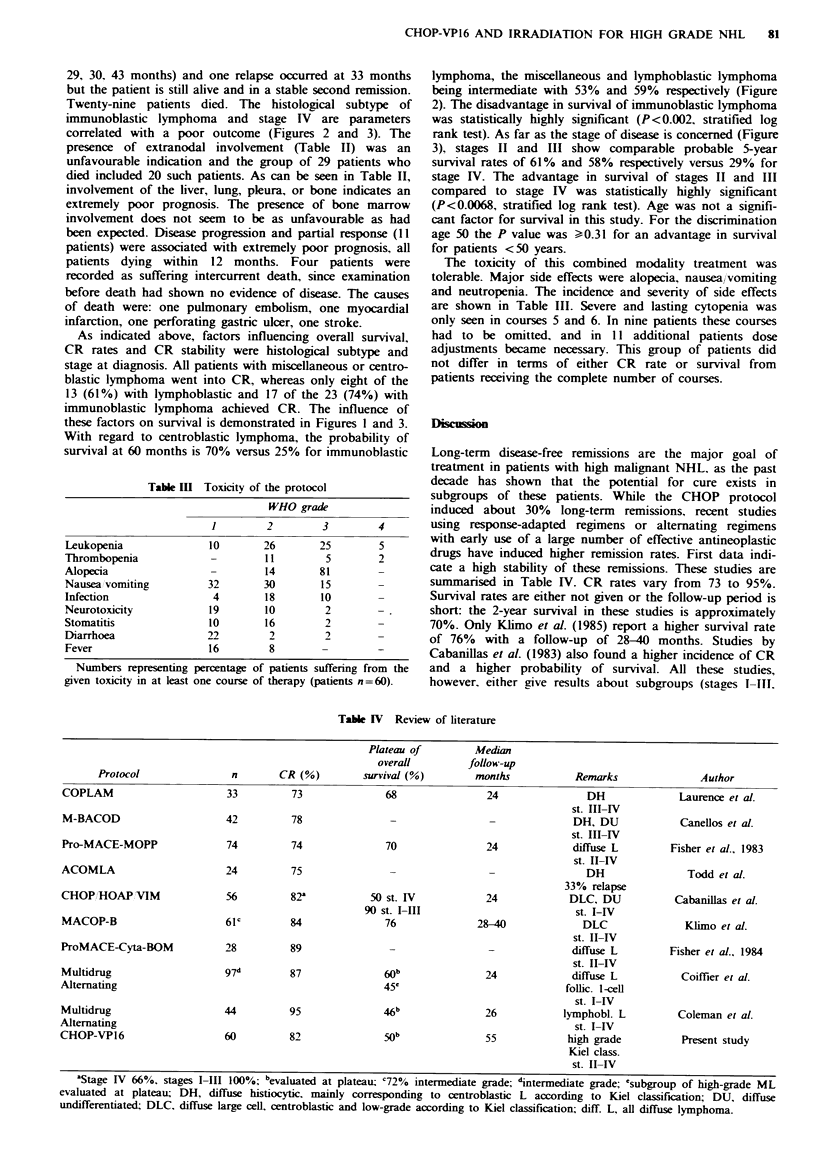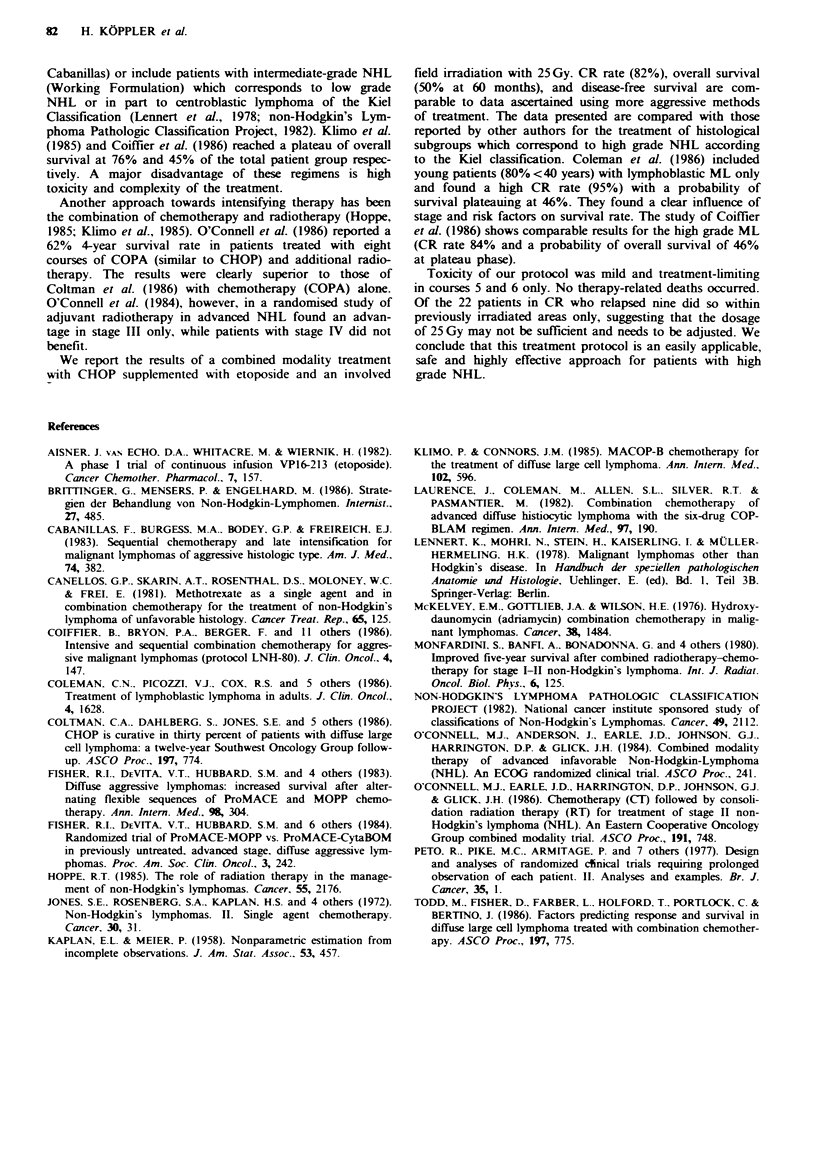# CHOP-VP16 chemotherapy and involved field irradiation for high grade non-Hodgkin's lymphomas: a phase II multicentre study.

**DOI:** 10.1038/bjc.1989.224

**Published:** 1989-07

**Authors:** H. KÃ¶ppler, K. H. PflÃ¼ger, I. Eschenbach, R. Pfab, K. Lennert, W. Wellens, M. Schmidt, W. D. Gassel, T. Kolb, R. HÃ¤ssler

**Affiliations:** Department of Internal Medicine, Philipps-University, Marburg, Federal Republic of Germany.

## Abstract

Sixty previously untreated patients with high grade non-Hodgkin's lymphomas stages II-IV received cyclophosphamide 750 mg m2 i.v., doxorubicin 50 mg m2 i.v., and vincristine 2 mg i.v. on day 1, prednisolone 100 mg p.o. on days 1-5 and etoposide 100 mg m2 i.v. on days 3-5 (CHOP-VP16). After four courses an involved field irradiation with a total dose of 25 Gy was employed and followed by two additional courses of CHOP-VP16. The overall response rate was 93%, with 49 patients (82%) achieving a complete remission (CR). Seven patients had a partial response and four patients showed no response. During a median follow-up period of 55 months, 22 of the 49 patients with CR relapsed, seven of them achieving a second complete remission with the same drug regimen. A maintained complete remission of up to 68 months was seen in 55% of all patients initially achieving CR. The median survival is 43 months. Mean side-effects of this drug regimen were alopecia (89%), nausea/vomiting (76%) and leukopenia (61%). No therapy-related deaths were seen. The results of this study demonstrate that this combined modality treatment produces high complete remission rates and that more than half of these patients achieve long-term disease-free survival.


Br. J. Cancer (1989), 60, 79-82 © The Macmillan Press Ltd., 1989

### CHOP-VP16 chemotherapy and involved field irradiation for high grade non-Hodgkin's lymphomas: a phase II multicentre study

H. Köppler[1], K.H. Pflüger[1], I. Eschenbach[1], R. Pfab[2], K. Lennert[3], W. Wellens[10], M. Schmidt[4], W.D. Gassel[5], T. Kolb[6], R. Häßler[7], K. Schumacher[8], G. v. Speth[9], R. Holle[11] & K. Havemann[1]

[1]*Department of Internal Medicine, Division of Haematology/Oncology, Philipps-University, Baldingerstrasse, D-3550 Marburg,* [2]*Division of Radiology, Philipps-University, Marburg,* [3]*Institute of Pathology/Anatomy, University of Kiel,* [4]*Evangelische Diakonissen-Anstalt, Internal Medicine, Bremen,* [5]*Städtisches Krankenhaus, Division Haematology/Oncology, Passau,* [6]*Hospital St Joseph-Stift, Bremen,* [7]*Städtische Kliniken, Internal Medicine I, Fulda,* [8]*Robert-Bosch-Hospital, Internal Medicine, Stuttgart,* [9]*Rot-Kreuz-Hospital, Internal Medicine, Bremen,* [10]*Krankenhaus der Barmherzigen Brueder, Dept of Oncology, Regensburg,* [11]*Biostatistics and Data Centre (ZMBT), University of Heidelberg, Federal Republic of Germany.*

**Summary** Sixty previously untreated patients with high grade non-Hodgkin's lymphomas stages II-IV received cyclophosphamide 750 mg m$^{2}$ i.v., doxorubicin 50 mg m$^{2}$ i.v., and vincristine 2 mg i.v. on day 1, prednisolone 100 mg p.o. on days 1-5 and etoposide 100 mg m$^{2}$ i.v. on days 3-5 (CHOP-VP16). After four courses an involved field irradiation with a total dose of 25 Gy was employed and followed by two additional courses of CHOP-VP16. The overall response rate was 93%, with 49 patients (82%) achieving a complete remission (CR). Seven patients had a partial response and four patients showed no response. During a median follow-up period of 55 months, 22 of the 49 patients with CR relapsed, seven of them achieving a second complete remission with the same drug regimen. A maintained complete remission of up to 68 months was seen in 55% of all patients initially achieving CR. The median survival is 43 months. Mean side-effects of this drug regimen were alopecia (89%), nausea/vomiting (76%) and leukopenia (61%). No therapy-related deaths were seen. The results of this study demonstrate that this combined modality treatment produces high complete remission rates and that more than half of these patients achieve long-term disease-free survival.

One of the major objectives of most current therapeutic trials in high grade non-Hodgkin's lymphomas (NHL) is to devise a form of treatment that will consistently induce a high frequency of complete remissions and ultimately benefit more patients in terms of longer disease-free survival. With CHOP, the standard protocol of the past decade introduced by McKelvey *et al.* (1976), complete remissions can be obtained in 50-60% of all patients with high grade NHL. Approximately half of these patients will eventually relapse with a poor prognosis. Recent results of studies applying a response-adapted chemotherapy (Fisher *et al.*, 1984) or an intensive chemotherapy with an alternating regimen (Brittinger *et al.*, 1986; Cabanillas *et al.*, 1983; Canellos *et al.*, 1981; Hoppe, 1985; Laurence *et al.*, 1982; Todd *et al.*, 1986) have shown higher complete remission rates which seem to be more stable. Due to the complexity of these protocols they have not been used extensively. In 1982 we initiated a multicentre phase II trial with an easily applicable protocol using the initial CHOP plus VP16 which has been shown to be an effective single agent in NHL (Aisner *et al.*, 1982; Jones *et al.*, 1972). Additionally patients received an involved field irradiation. The results of this study are presented here.

### Patients and methods

### *Patients*

Sixty previously untreated patients with high grade NHL stages II-IV entered the study. Patient eligibility included: histologically confirmed high grade NHL (Kiel classification (Lennert *et al.*, 1978)); no prior chemotherapy; no other malignancy; stages II-IV (Ann Arbor classification). Patients with lymphoblastic NHL aged <25 years were excluded from this study.

Patients' data in terms of age, sex, Karnofsky index, stage and histological subtype are shown in Table I. Median age

**Table I** Patients' data

| | | *n* | % |
|---|---|---|---|
| Age | >50 | 39 | 65 |
| | ≤50 | 21 | 35 |
| Sex | Male | 30 | 50 |
| | Female | 30 | 50 |
| Karnofsky | >80% | 48 | 80 |
| | ≤80% | 12 | 20 |
| Stage | II | 20 | 33 |
| | III | 17 | 28 |
| | IV | 23 | 39 |
| B-symptoms | | 20 | 33 |
| Histology | Centroblastic NHL | 17 | 28 |
| | Immunoblastic NHL | 23 | 38 |
| | Lymphoblastic NHL | 13 | 22 |
| | Miscellaneous NHL | 7 | 12 |

*Miscellaneous NHL*
3 Kil-ML (1 T-type, 2 B-type)
3 undifferentiated ML
1 histiocytic ML

**Table II** Incidence of extranodal involvement and clinical outcome

| *Localisation* | *n* | *Alive* | *Death* |
|---|---|---|---|
| Bone marrow | 7 | 4 | 3 |
| Liver | 7 | – | 7 |
| Pleura | 5 | 1 | 4 |
| Lung | 4 | 1 | 3 |
| Bone | 4 | 1 | 3 |
| Tonsil | 4 | 4 | – |
| Nose/orbit | 3 | 1 | 2 |
| GI tract | 3 | 2 | 1 |
| Skin | 3 | 1 | 2 |
| Pericardium | 3 | – | 3 |
| Ovary/uterus | 2 | – | 2 |
| Muscle | 1 | 1 | – |
| Kidney | 1 | – | 1 |
| Pancreas | 1 | – | 1 |

Correspondence: H. Köppler.
Received 6 June 1988, and accepted in revised form 14 December 1988.

was 56 years (range 15–73 years). Three patients were under 30 years of age, 65% were over 50 and 40% over 60 years. The details of extranodal involvement are summarised in Table II.

*Histological classification*

Lymph node material after diagnosis of high grade NHL by local pathologists was reviewed by Dr Karl Lennert or his associates at the University of Kiel, Institute of Pathology, and classified according to the criteria of Kiel classification (Lennert *et al.*, 1978). Immunophenotyping was performed whenever possible and only the University of Kiel opinion on histology was accepted.

*Staging procedures*

All patients underwent the following staging procedures: chest X-ray p.a. and lateral; bone-scan; abdominal CAT-scan and/or sonography; bilateral bone marrow biopsies of the posterior iliac crest; lumbar puncture in patients with lymphoblastic NHL; biochemical tests: AST, ALT, LDH, alkaline phosphatase, bilirubine, creatinine, potassium, sodium, calcium, CBC and platelet count.

*Chemotherapy*

Patients were treated with six courses of the following regimen: cyclophosphamide 750 mg m$^{-2}$ i.v., doxorubicin 50 mg m$^{-2}$ i.v. and vincristine 2 mg i.v. on day 1, prednisolone 100 mg p.o. on days 1–5, etoposide 100 mg m$^{-2}$ i.v. on days 3–5. Between courses 4 and 5 an involved field irradiation with a dose of 25 Gy was employed. The regimen was administered every 21 days. If cytopenia (WBC < 3,000 mm$^{-3}$ or platelet count of < 100,000 mm$^{-3}$) was present on day 21, therapy was delayed for a maximum of one week. A dose-adjusted course was given then. Patients with lymphoblastic NHL received an intrathekal CNS-prophylaxis with 15 mg methotrexate on days 1 and 5 in courses 1 and 2 and additional brain irradiation with 25 Gy after course 4.

*Radiotherapy*

Twenty-one days after course 4 all patients in CR or PR received an involved field irradiation with a total dosage of 25 Gy in 10–12 single fractions. Patients with persisting extranodal involvement were excluded. Irradiation was given to all nodal involvements and gastrointestinal lesions. Patients with lymphoblastic NHL received prophylactic CNS irradiation with 25 Gy after course 4. In patients with meningeal involvement CNS irradiation was given during course 1.

*Evaluation of response*

A complete restaging was performed after course 4, after involved field irradiation and after course 6. Complete remission (CR) required that all clinically, imaging or biopsy detectable tumours had disappeared. Partial response (PR) was defined as a reduction of ⩾50% in all measurable tumours and the absence of new lesions. No response was defined as less than 50% reduction of measurable lesions, no change or progress of the disease.

*Statistical analyses*

Survival curves were calculated and plotted by the actuarial method of Kaplan & Meier (1958). The significance of difference in survival of subgroups, taking into account the prognostic factors stage at diagnosis, histological subtype and age, was analysed by the stratified version of the log rank test (Peto *et al.*, 1977).

### Results

Between March 1982 and December 1985, 60 patients entered the protocol. All of them were evaluated before treatment and judged acceptable on the basis of the criteria outlined above. A complete remission was achieved in 49 patients (82%). Seven patients had a partial response and four patients had no response.

Of the patients who achieved CR, 75% did so after four courses of chemotherapy, the remaining achieved CR after radiotherapy. No additional CR was seen after courses 5 and 6. During the follow-up period (24–69 months, median 55 months) 20 patients with CR relapsed after 1–16 months. Two additional patients had a late relapse after 20 and 33 months respectively. In 20 patients the fifth and/or sixth chemotherapy cycle was omitted or dose-adjusted due to severe neutropenia after radiotherapy. Twelve of the patients in this subgroup are still well and in CR. Eight patients experienced a relapse and died. With a median follow-up of 55 months (24–69 months) the overall survival shows a plateau at almost 50%, as illustrated in Figure 1. Only four patients died after more than 16 months of observation (24,

**Figure 1** Overall survival (——) including all causes of death and event-free survival (– – –), event defined as death or relapse, of the 60 patients.

**Figure 2** Overall survival for subgroups of the 60 patients according to histological subtype (Kiel classification). The advantage in survival for centroblastic (–·–·–, $n = 17$), lymphoblastic (– – –, $n = 13$) and miscellaneous (. . . ., $n = 7$) as compared to immunoblastic lymphomas (——, $n = 23$) was statistically significant ($P < 0.002$).

**Figure 3** Overall survival for the 60 patients using Ann Arbor stage. The advantage in survival for stages II (– – –, $n = 20$) and III (. . . ., $n = 17$) as compared to stage IV (——, $n = 23$) was statistically significant ($P < 0.0068$).

29, 30, 43 months) and one relapse occurred at 33 months but the patient is still alive and in a stable second remission. Twenty-nine patients died. The histological subtype of immunoblastic lymphoma and stage IV are parameters correlated with a poor outcome (Figures 2 and 3). The presence of extranodal involvement (Table II) was an unfavourable indication and the group of 29 patients who died included 20 such patients. As can be seen in Table II, involvement of the liver, lung, pleura, or bone indicates an extremely poor prognosis. The presence of bone marrow involvement does not seem to be as unfavourable as had been expected. Disease progression and partial response (11 patients) were associated with extremely poor prognosis, all patients dying within 12 months. Four patients were recorded as suffering intercurrent death, since examination before death had shown no evidence of disease. The causes of death were: one pulmonary embolism, one myocardial infarction, one perforating gastric ulcer, one stroke.

As indicated above, factors influencing overall survival, CR rates and CR stability were histological subtype and stage at diagnosis. All patients with miscellaneous or centroblastic lymphoma went into CR, whereas only eight of the 13 (61%) with lymphoblastic and 17 of the 23 (74%) with immunoblastic lymphoma achieved CR. The influence of these factors on survival is demonstrated in Figures 1 and 3. With regard to centroblastic lymphoma, the probability of survival at 60 months is 70% versus 25% for immunoblastic lymphoma, the miscellaneous and lymphoblastic lymphoma being intermediate with 53% and 59% respectively (Figure 2). The disadvantage in survival of immunoblastic lymphoma was statistically highly significant ($P<0.002$, stratified log rank test). As far as the stage of disease is concerned (Figure 3), stages II and III show comparable probable 5-year survival rates of 61% and 58% respectively versus 29% for stage IV. The advantage in survival of stages II and III compared to stage IV was statistically highly significant ($P<0.0068$, stratified log rank test). Age was not a significant factor for survival in this study. For the discrimination age 50 the $P$ value was $\geqslant 0.31$ for an advantage in survival for patients $<50$ years.

The toxicity of this combined modality treatment was tolerable. Major side effects were alopecia, nausea/vomiting and neutropenia. The incidence and severity of side effects are shown in Table III. Severe and lasting cytopenia was only seen in courses 5 and 6. In nine patients these courses had to be omitted, and in 11 additional patients dose adjustments became necessary. This group of patients did not differ in terms of either CR rate or survival from patients receiving the complete number of courses.

**Table III** Toxicity of the protocol

| | *WHO grade* | | | |
|---|---|---|---|---|
| | *1* | *2* | *3* | *4* |
| Leukopenia | 10 | 26 | 25 | 5 |
| Thrombopenia | – | 11 | 5 | 2 |
| Alopecia | – | 14 | 81 | – |
| Nausea/vomiting | 32 | 30 | 15 | – |
| Infection | 4 | 18 | 10 | – |
| Neurotoxicity | 19 | 10 | 2 | – |
| Stomatitis | 10 | 16 | 2 | – |
| Diarrhoea | 22 | 2 | 2 | – |
| Fever | 16 | 8 | – | – |

Numbers representing percentage of patients suffering from the given toxicity in at least one course of therapy (patients $n=60$).

### Discussion

Long-term disease-free remissions are the major goal of treatment in patients with high malignant NHL, as the past decade has shown that the potential for cure exists in subgroups of these patients. While the CHOP protocol induced about 30% long-term remissions, recent studies using response-adapted regimens or alternating regimens with early use of a large number of effective antineoplastic drugs have induced higher remission rates. First data indicate a high stability of these remissions. These studies are summarised in Table IV. CR rates vary from 73 to 95%. Survival rates are either not given or the follow-up period is short: the 2-year survival in these studies is approximately 70%. Only Klimo *et al.* (1985) report a higher survival rate of 76% with a follow-up of 28–40 months. Studies by Cabanillas *et al.* (1983) also found a higher incidence of CR and a higher probability of survival. All these studies, however, either give results about subgroups (stages I–III,

**Table IV** Review of literature

| *Protocol* | *n* | *CR (%)* | *Plateau of overall survival (%)* | *Median follow-up months* | *Remarks* | *Author* |
|---|---|---|---|---|---|---|
| COPLAM | 33 | 73 | 68 | 24 | DH st. III–IV | Laurence *et al.* |
| M-BACOD | 42 | 78 | – | – | DH, DU st. III–IV | Canellos *et al.* |
| Pro-MACE-MOPP | 74 | 74 | 70 | 24 | diffuse L st. II–IV | Fisher *et al.*, 1983 |
| ACOMLA | 24 | 75 | – | – | DH 33% relapse | Todd *et al.* |
| CHOP/HOAP/VIM | 56 | 82[a] | 50 st. IV 90 st. I–III | 24 | DLC, DU st. I–IV | Cabanillas *et al.* |
| MACOP-B | 61[c] | 84 | 76 | 28–40 | DLC st. II–IV | Klimo *et al.* |
| ProMACE-Cyta-BOM | 28 | 89 | – | – | diffuse L st. II–IV | Fisher *et al.*, 1984 |
| Multidrug Alternating | 97[d] | 87 | 60[b] 45[e] | 24 | diffuse L follic. 1-cell st. I–IV | Coiffier *et al.* |
| Multidrug Alternating | 44 | 95 | 46[b] | 26 | lymphobl. L st. I–IV | Coleman *et al.* |
| CHOP-VP16 | 60 | 82 | 50[b] | 55 | high grade Kiel class. st. II–IV | Present study |

[a]Stage IV 66%, stages I–III 100%; [b]evaluated at plateau; [c]72% intermediate grade; [d]intermediate grade; [e]subgroup of high-grade ML evaluated at plateau; DH, diffuse histiocytic, mainly corresponding to centroblastic L according to Kiel classification; DU, diffuse undifferentiated; DLC, diffuse large cell, centroblastic and low-grade according to Kiel classification; diff. L, all diffuse lymphoma.

Cabanillas) or include patients with intermediate-grade NHL (Working Formulation) which corresponds to low grade NHL or in part to centroblastic lymphoma of the Kiel Classification (Lennert *et al.*, 1978; non-Hodgkin's Lymphoma Pathologic Classification Project, 1982). Klimo *et al.* (1985) and Coiffier *et al.* (1986) reached a plateau of overall survival at 76% and 45% of the total patient group respectively. A major disadvantage of these regimens is high toxicity and complexity of the treatment.

Another approach towards intensifying therapy has been the combination of chemotherapy and radiotherapy (Hoppe, 1985; Klimo *et al.*, 1985). O'Connell *et al.* (1986) reported a 62% 4-year survival rate in patients treated with eight courses of COPA (similar to CHOP) and additional radiotherapy. The results were clearly superior to those of Coltman *et al.* (1986) with chemotherapy (COPA) alone. O'Connell *et al.* (1984), however, in a randomised study of adjuvant radiotherapy in advanced NHL found an advantage in stage III only, while patients with stage IV did not benefit.

We report the results of a combined modality treatment with CHOP supplemented with etoposide and an involved field irradiation with 25 Gy. CR rate (82%), overall survival (50% at 60 months), and disease-free survival are comparable to data ascertained using more aggressive methods of treatment. The data presented are compared with those reported by other authors for the treatment of histological subgroups which correspond to high grade NHL according to the Kiel classification. Coleman *et al.* (1986) included young patients (80% <40 years) with lymphoblastic ML only and found a high CR rate (95%) with a probability of survival plateauing at 46%. They found a clear influence of stage and risk factors on survival rate. The study of Coiffier *et al.* (1986) shows comparable results for the high grade ML (CR rate 84% and a probability of overall survival of 46% at plateau phase).

Toxicity of our protocol was mild and treatment-limiting in courses 5 and 6 only. No therapy-related deaths occurred. Of the 22 patients in CR who relapsed nine did so within previously irradiated areas only, suggesting that the dosage of 25 Gy may not be sufficient and needs to be adjusted. We conclude that this treatment protocol is an easily applicable, safe and highly effective approach for patients with high grade NHL.

**References**

AISNER, J. VAN ECHO, D.A., WHITACRE, M. & WIERNIK, H. (1982). A phase I trial of continuous infusion VP16-213 (etoposide). *Cancer Chemother. Pharmacol.*, **7**, 157.

BRITTINGER, G., MENSERS, P. & ENGELHARD, M. (1986). Strategien der Behandlung von Non-Hodgkin-Lymphomen. *Internist.*, **27**, 485.

CABANILLAS, F., BURGESS, M.A., BODEY, G.P. & FREIREICH, E.J. (1983). Sequential chemotherapy and late intensification for malignant lymphomas of aggressive histologic type. *Am. J. Med.*, **74**, 382.

CANELLOS, G.P., SKARIN, A.T., ROSENTHAL, D.S., MOLONEY, W.C. & FREI, E. (1981). Methotrexate as a single agent and in combination chemotherapy for the treatment of non-Hodgkin's lymphoma of unfavorable histology. *Cancer Treat. Rep.*, **65**, 125.

COIFFIER, B., BRYON, P.A., BERGER, F. and 11 others (1986). Intensive and sequential combination chemotherapy for aggressive malignant lymphomas (protocol LNH-80). *J. Clin. Oncol.*, **4**, 147.

COLEMAN, C.N., PICOZZI, V.J., COX, R.S. and 5 others (1986). Treatment of lymphoblastic lymphoma in adults. *J. Clin. Oncol.*, **4**, 1628.

COLTMAN, C.A., DAHLBERG, S., JONES, S.E. and 5 others (1986). CHOP is curative in thirty percent of patients with diffuse large cell lymphoma: a twelve-year Southwest Oncology Group follow-up. *ASCO Proc.*, **197**, 774.

FISHER, R.I., DEVITA, V.T., HUBBARD, S.M. and 4 others (1983). Diffuse aggressive lymphomas: increased survival after alternating flexible sequences of ProMACE and MOPP chemotherapy. *Ann. Intern. Med.*, **98**, 304.

FISHER, R.I., DEVITA, V.T., HUBBARD, S.M. and 6 others (1984). Randomized trial of ProMACE-MOPP vs. ProMACE-CytaBOM in previously untreated, advanced stage, diffuse aggressive lymphomas. *Proc. Am. Soc. Clin. Oncol.*, **3**, 242.

HOPPE, R.T. (1985). The role of radiation therapy in the management of non-Hodgkin's lymphomas. *Cancer*, **55**, 2176.

JONES, S.E., ROSENBERG, S.A., KAPLAN, H.S. and 4 others (1972). Non-Hodgkin's lymphomas. II. Single agent chemotherapy. *Cancer*, **30**, 31.

KAPLAN, E.L. & MEIER, P. (1958). Nonparametric estimation from incomplete observations. *J. Am. Stat. Assoc.*, **53**, 457.

KLIMO, P. & CONNORS, J.M. (1985). MACOP-B chemotherapy for the treatment of diffuse large cell lymphoma. *Ann. Intern. Med.*, **102**, 596.

LAURENCE, J., COLEMAN, M., ALLEN, S.L., SILVER, R.T. & PASMANTIER, M. (1982). Combination chemotherapy of advanced diffuse histiocytic lymphoma with the six-drug COP-BLAM regimen. *Ann. Intern. Med.*, **97**, 190.

LENNERT, K., MOHRI, N., STEIN, H., KAISERLING, I. & MÜLLER-HERMELING, H.K. (1978). Malignant lymphomas other than Hodgkin's disease. In *Handbuch der speziellen pathologischen Anatomie und Histologie*, Uehlinger, E. (ed), Bd. 1, Teil 3B. Springer-Verlag: Berlin.

MCKELVEY, E.M., GOTTLIEB, J.A. & WILSON, H.E. (1976). Hydroxydaunomycin (adriamycin) combination chemotherapy in malignant lymphomas. *Cancer*, **38**, 1484.

MONFARDINI, S., BANFI, A., BONADONNA, G. and 4 others (1980). Improved five-year survival after combined radiotherapy–chemotherapy for stage I–II non-Hodgkin's lymphoma. *Int. J. Radiat. Oncol. Biol. Phys.*, **6**, 125.

NON-HODGKIN'S LYMPHOMA PATHOLOGIC CLASSIFICATION PROJECT (1982). National cancer institute sponsored study of classifications of Non-Hodgkin's Lymphomas. *Cancer*, **49**, 2112.

O'CONNELL, M.J., ANDERSON, J., EARLE, J.D., JOHNSON, G.J., HARRINGTON, D.P. & GLICK, J.H. (1984). Combined modality therapy of advanced infavorable Non-Hodgkin-Lymphoma (NHL). An ECOG randomized clinical trial. *ASCO Proc.*, 241.

O'CONNELL, M.J., EARLE, J.D., HARRINGTON, D.P., JOHNSON, G.J. & GLICK, J.H. (1986). Chemotherapy (CT) followed by consolidation radiation therapy (RT) for treatment of stage II non-Hodgkin's lymphoma (NHL). An Eastern Cooperative Oncology Group combined modality trial. *ASCO Proc.*, **191**, 748.

PETO, R., PIKE, M.C., ARMITAGE, P. and 7 others (1977). Design and analyses of randomized clinical trials requiring prolonged observation of each patient. II. Analyses and examples. *Br. J. Cancer*, **35**, 1.

TODD, M., FISHER, D., FARBER, L., HOLFORD, T., PORTLOCK, C. & BERTINO, J. (1986). Factors predicting response and survival in diffuse large cell lymphoma treated with combination chemotherapy. *ASCO Proc.*, **197**, 775.